# High-Mobility All-Transparent TFTs with Dual-Functional Amorphous IZTO for Channel and Transparent Conductive Electrodes

**DOI:** 10.3390/ma18020216

**Published:** 2025-01-07

**Authors:** Min-Woo Park, Sohyeon Kim, Su-Yeon Son, Si-Won Kim, Tae-Kyun Moon, Pei-Chen Su, Kyoung-Kook Kim

**Affiliations:** 1 Department of IT Semiconductor Convergence Engineering, Research Institute of Advanced Convergence Technology, Tech University of Korea, Siheung 15073, Republic of Korea; 2 Department of Nano & Semiconductor Engineering, Tech University of Korea, Siheung 15073, Republic of Korea; 3 School of Mechanical and Aerospace Engineering, Nanyang Technological University, 50 Nanyang Avenue, Singapore 639798, Singapore

**Keywords:** a-IZTO, all-transparent TFT, amorphous oxide TFT, flexible device, transparent display

## Abstract

The increasing demand for advanced transparent and flexible display technologies has led to significant research in thin-film transistors (TFTs) with high mobility, transparency, and mechanical robustness. In this study, we fabricated all-transparent TFTs (AT-TFTs) utilizing amorphous indium-zinc-tin-oxide (a-IZTO) as a dual-functional material for both the channel layer and transparent conductive electrodes (TCEs). The a-IZTO was deposited using radio-frequency magnetron sputtering, with its composition adjusted for both channel and electrode functionality. XRD analysis confirmed the amorphous nature of the a-IZTO layers, ensuring structural stability post-thermal annealing. The a-IZTO TCEs demonstrated high optical transparency (89.57% in the visible range) and excellent flexibility, maintaining a low sheet resistance with minimal degradation even after 100,000 bending cycles. The fabricated AT-TFTs exhibit superior field-effect mobility (30.12 cm^2^/V·s), an on/off current ratio exceeding 10^8^, and a subthreshold swing of 0.36 V/dec. The AT-TFT device demonstrated a minimum transmittance of 75.46% in the visible light range, confirming its suitability for next-generation flexible and transparent displays.

## 1. Introduction

The demand for advanced display devices is steadily increasing due to technological advancements [1]. Transparent displays are used for various purposes, such as mobile devices, head-up displays, and large-scale screens, because of their optical properties. All layers of transparent displays need to have higher transmittance in the visible light range to achieve excellent clarity. This means that not only the light-emitting layer of the display but also the thin film transistor (TFT) for display driving must exhibit transparent properties in the visible light range. In addition, transparent displays are applied to physically deformable devices such as foldable devices, rollable devices, and stretchable devices. As a result, much research is being conducted on transparent and flexible TFTs.

Amorphous oxide semiconductors (AOSs), with several advantages, including high carrier mobility, low growth temperature, excellent uniformity, and high transparency in the visible range, have been attractive as a TFT material for next-generation display [2,3]. Among many AOSs, many studies have recently been reported on amorphous indium-gallium-zinc-oxide (a-IGZO), which is the AOS material most commonly used as a TFT channel layer [4,5,6]. However, the field effect mobility of a-IGZO TFTs is in the range of 10–20 cm^2^/V·s, and it is not high enough (≥30 cm^2^/V·s) for next-generation display applications. Amorphous indium-zinc-tin-oxide (a-IZTO), which has higher field-effect mobility higher than 30 cm^2^/V·s, is attractive as an alternative material of a-IGZO for TFTs. C.-S. Fuh et al. reported high performance of a-IZTO TFT with field-effect mobility of 39.6 cm^2^/V·s, threshold voltage (Vth) of −2.8 V, and subthreshold swing of 0.25 V/dec via thermal annealing [7]. P.-T. Liu et al. reported reliable a-IZTO TFT with field-effect mobility of 30.16 cm^2^/V·s, Vth of −0.88 V, and subthreshold swing of 0.34 V/dec via Al_2_O_3_ passivation [8].

However, most AOS TFTs, including a-IZTO TFTs, generally have only the channel layer made of AOSs. Previous research on AOS TFTs has shown that crystalline or metallic thin films are mainly used as transparent conductive electrodes (TCEs) for the source, drain, and gate of AOS TFTs. TCEs are defined under specific conditions where the electric resistivity ranges from 10^−4^ to 10^−3^ Ω·cm, and the optical transmittance is over 80% in the visible light range [9]. In the case of metallic thin film for TCEs, it can be transparent if the thickness is thin enough, like a few nanometers. However, such thin metal layers have poor electric properties and physical stability [10,11]. Hence, there is a limitation in applying transparent displays. Meanwhile, in the case of crystalline TCEs, they need stable electrical performance, which benefits from the advantage of electron mobility in crystalline structures such as indium tin oxide (ITO) and fluorine tin oxide (FTO) [12]. To make thin films with crystalline structures, they need to be exposed to high temperatures to gain enough kinetic energy for atom diffusion. To achieve high performance, an annealing process at a temperature over 500 is required, which complicates fabrication and increases costs [13,14,15]. Additionally, thin films composed of crystalline structures are prone to damage when subjected to bending or flexing. To prevent breakage, crystalline thin films must be grown to a very small thickness, resulting in films that are too thin to attain the appropriate level of electrical resistance. These drawbacks limit its application to transparent and flexible displays. Meanwhile, AOS thin films demonstrate high transparency in the visible range and stable electrical and physical performance when bent, particularly at a greater thickness compared to crystalline thin films [16]. With controlled characteristics, AOS thin films can be utilized not only as the channel layer of TFTs but also as TCEs. Despite this potential, there has been no reported research on TCEs using amorphous materials yet. Among AOS materials, the commonly used a-IGZO contains Ga cations that suppress the generation of electron carriers, making it challenging to achieve sufficient conductivity for use as an electrode, even with compositional adjustments [17]. In contrast, a-IZTO can attain the required conductivity for electrode applications by modifying its composition. Therefore, we selected a-IZTO as TCEs for AOS TFTs.

In this study, we fabricated all-transparent TFTs (AT-TFTs) that can be used in flexible devices by using a-IZTO material for the TCEs. The a-IZTO material serves a dual function as both the channel layer and the TCEs in the AT-TFTs. We changed the composition of the a-IZTO to define the properties of the channel layer and TCEs. The dual-functional a-IZTO was deposited using the radio-frequency (RF) magnetron sputtering process to control the composition. The AT-TFTs with the dual-functional a-IZTO showed significantly high performance in all aspects of transfer properties, including field-effect mobility (30.12 cm^2^/V·s), on/off ratio (~10^9^), and low subthreshold swing (0.36 V/dec).

## 2. Materials and Methods

### 2.1. Thin Film Deposition

Dual-functional a-IZTO was deposited using RF magnetron sputtering. We used two compound single targets with different molar concentrations, each composed of three elements: In, Zn, and Sn. The molar composition ratios of the target were 55:37:8 at% for the channel layer and 76:20:4 at% for the source, drain, and bottom gate, which served as TCEs. During the sputtering process, the base pressure was maintained at 1 × 10^−7^ Torr (1.33 × 10^−5^ Pa), with a substrate-target distance of 7 cm and a working pressure of 3 mTorr. To investigate the characteristics of each a-IZTO for channel and TCEs, the single layer of a-IZTO was deposited. For a-IZTO for the channel layer, a-IZTO was deposited onto a sapphire substrate, while for the TCEs, it was deposited on both sapphire substrates and polyimide (PI) films. The RF power for the deposition of a-IZTO as TCEs was varied at 30, 50, 70, and 90 W.

### 2.2. AT-TFT Fabrication

**Figure 1:**
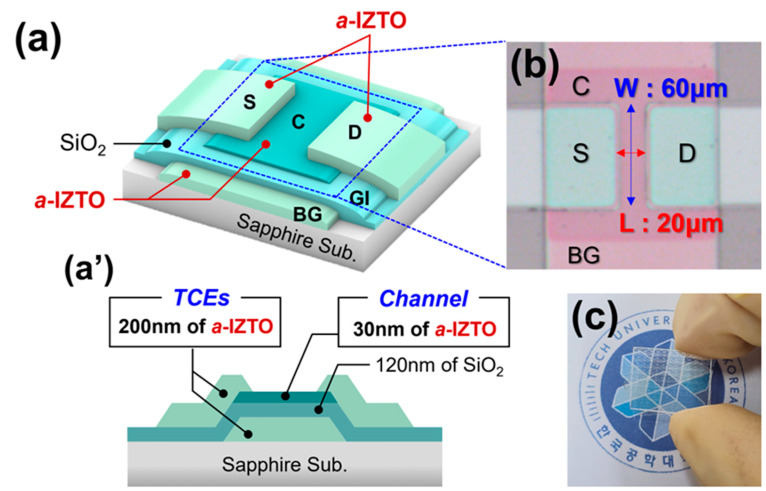
(**a**) The three-dimensional schematic image of the AT-TFT device using amorphous SiO_2_ and dual-functional a-IZTO for channel and TCEs. (**a’**) The cross-sectional schematic image of the AT-TFT device. (**b**) The top view image of AT-TFT which has 60 μm of channel width and 20 μm of channel length. (**c**) The image of the fabricated AT-TFT wafer which has high transparency.

Figure 1a shows the schematic of an AT-TFT device, which is entirely fabricated using amorphous oxide materials. The channel, source, drain, and bottom gate are composed of a-IZTO, except for the SiO_2_ used as the gate insulator in the AT-TFTs, as shown in Figure 1a’. The AT-TFTs were fabricated on a sapphire substrate, which underwent a cleaning process involving ultrasonic treatment in acetone, isopropanol (IPA), and ethanol for 5 min each. This was followed by rinsing in deionized (DI) water (with a resistivity ≥ 18.3 MΩ-cm) and drying with compressed N_2_ gas. First, a 200 nm thick layer of a-IZTO was deposited onto the sapphire substrate to form the bottom gate electrode of the AT-TFT using sputtering deposition. Then, 120 nm of SiO_2_ was deposited as the gate insulator on the gate electrode using plasma-enhanced chemical vapor deposition (PECVD). Next, a 30 nm thick layer of a-IZTO was deposited to serve as the channel layer. The channel width (W) and length (L) were set at 60 µm and 20 µm, respectively (W/L ratio = 3), as depicted in Figure 1b. After the channel layer deposition, thermal annealing was performed in a box furnace at 300 °C for 15 min to activate the dopants in the channel layer. Subsequently, source and drain electrodes, also with a thickness of 200 nm, were deposited using a-IZTO under the same sputtering conditions as the bottom gate. Then, the gate insulator was etched using a buffered oxide etchant (HF:DI = 1:6) in a wet etch process to apply a gate voltage to the gate electrode. Figure 1c displays the fabricated AT-TFT wafer, which exhibits high transparency.

### 2.3. AT-TFT Characterization

The electrical properties of the a-IZTO for TCEs were characterized using Hall effect measurements (HL5500, Nanometrics, Kanata, CA, USA). The metal composition of the a-IZTO for the channel and TCEs was analyzed by scanning electron microscope energy dispersive spectrometry (SEM-EDS, S4300, Hitachi Ltd., Tokyo, Japan). Bending tests were conducted on the a-IZTO for TCEs deposited on 180 μm-thick PI film to evaluate the flexibility of the amorphous electrodes and any changes in electrical properties due to mechanical movement. A U-shaped bending test machine with a bending radius of 3 mm was employed, and the bending cycles were set at 20,000, 40,000, 60,000, 80,000, and 100,000. To confirm the crystal structure of a-IZTO for channel and TCEs, we utilized an X-ray diffractometer (XRD, X’pert Pro MPD, Malvern PANalytical, Malvern, UK). The transmittance of the channel and TCE films was measured using a UV-visible spectrophotometer (Cary 300 Bio, Varian, Palo Alto, CA, USA). The output and transfer curves of the AT-TFT were measured using a source meter (4200A-SCS parameter analyzer, Keithley Instruments Inc., Solon, OH, USA).

## 3. Results and Discussion

**Figure 2:**
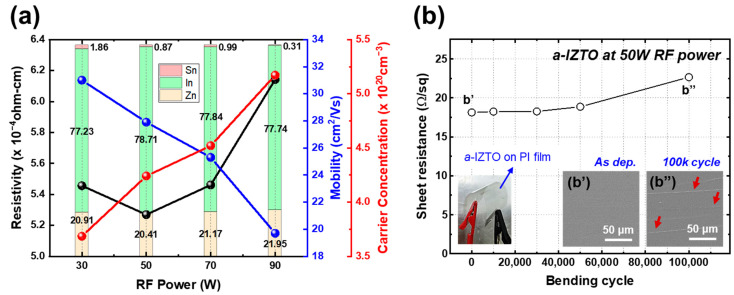
(**a**) The resistivity, mobility, carrier concentration, and metal atom proportion of a-IZTO for TCEs deposited by RF magnetron sputtering with RF power ranging from 30 to 90 W. The black line, blue line, and red line represent the resistivity, carrier mobility, and carrier concentration of a-IZTO for TCEs, respectively. (**b**) Bending test results of a-IZTO for TCE deposited by sputtering at RF power of 50 W (bending cycles = 0, 20,000, 40,000, 60,000, 80,000, 100,000). The inset image within the graph shows the flexible bending of the a-IZTO deposited on the PI film. The FE-SEM images of (**b’**) the as-deposited a-IZTO surface and (**b”**) the a-IZTO surface after 100,000 bending cycles. The red arrow in (**b”**) indicates the cracks on the a-IZTO surface after 100,000 bending cycles.

Figure 2 shows the electrical properties and bending test results of the a-IZTO film used for TCEs. In Figure 2a, the resistivity, carrier concentration, and mobility of a-IZTO are presented in relation to the proportion of metal atoms within the film. The bar graph in the background of Figure 2a indicates the varying concentrations of metal atoms in the a-IZTO thin films at different RF power levels. During the sputtering process, the sputtering yield of ZnO was lower than that of In_2_O_3_ due to the higher surface stability of the ZnO target compared to In_2_O_3_. As the RF power increased, the density of the plasma generated by ionized argon (Ar) gas rose. This increase allows Ar^+^ ions to gain sufficient energy to dislodge ZnO crystals from the surface of the IZTO target. Consequently, the Zn concentration in the film increases with higher RF power. We observed that the mobility of the a-IZTO decreased while its carrier concentration increased. This change displayed an approximately linear trend with rising RF power. When RF power increased, the energy of the sputtered particles from the target surface also increased. These high-energy particles not only contribute to a more efficient deposition process but also allow for enhanced surface migration on the substrate, facilitating the formation of denser, thin films. This increased density reduces the voids and defects that typically limit electrical conductivity, thereby increasing the carrier concentration in the film. However, the increased carrier concentration also creates additional scattering centers, leading to decreased carrier mobility. As a result, the a-IZTO deposited at 50 W RF power exhibited the lowest resistance. The resistivity, carrier concentration, and mobility of a-IZTO for the channel could not be measured because its conductivity was insufficient for the Hall effect measurement. The metal composition of a-IZTO for the channel was analyzed in Table S1 to determine whether its composition is suitable for use as a channel layer.

Figure 2b shows the bending test results with a bending radius of 3 mm conducted to evaluate the flexibility and electrical performance of a-IZTO deposited at the RF power of 50 W. Table S2 displays the surface resistance values corresponding to the number of bending cycles and the changes in surface resistance relative to the as-deposited a-IZTO. The as-deposited a-IZTO demonstrated excellent flexibility and a low sheet resistance of 18.13 Ω/sq. Notably, the increase in sheet resistance remained minimal, below 4%, even after 50,000 bending cycles. After 100,000 bending cycles, the sheet resistance of a-IZTO was recorded at 22.65 Ω/sq, indicating a 24.95% increase compared to the as-deposited a-IZTO. Figure 2b’,b” depict the SEM images of the as-deposited a-IZTO and the a-IZTO after 100,000 bending cycles, respectively. In Figure 2b’, the surface of the a-IZTO appears defect-free, while in Figure 2b”, cracking due to compressive stress is confirmed. This observation suggests that the degradation in sheet resistance characteristics is attributed to the cracks that developed on the surface of the a-IZTO following the 100,000 bending test. The sheet resistance variation under a 3 mm bending radius is remarkably stable compared to the previous studies [18,19]. Furthermore, as shown in Figure S1, no change in sheet resistance was observed even after 100,000 bending cycles at a 10 mm bending radius. These results confirm that a-IZTO TCEs exhibit outstanding properties, making them highly suitable for application in flexible devices.

**Figure 3:**
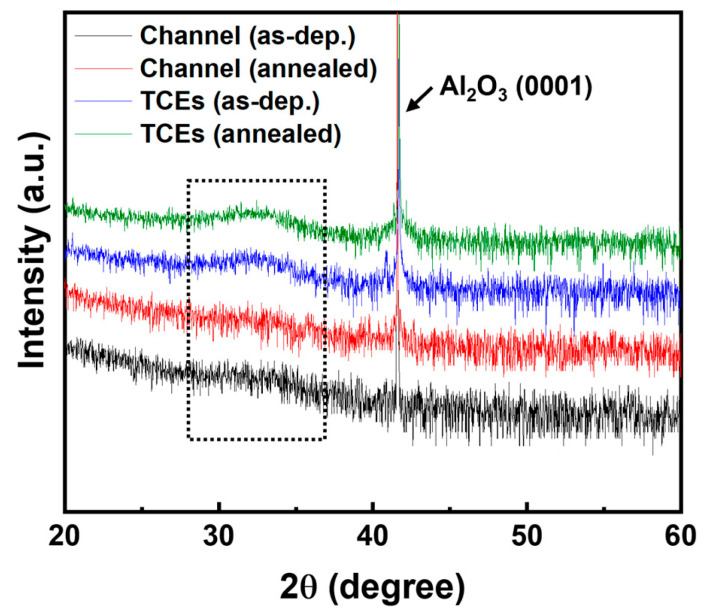
The XRD results of dual-functional a-IZTO films for 30 nm-thick channels and 200 nm-thick TCEs before and after a thermal annealing process at 300 °C for 15 min. The broad peaks in the black box indicate that both IZTO for the channel and TCEs are in an amorphous state.

To evaluate the structural properties of a-IZTO used as both the TCEs and channel layer, XRD analysis was performed, as shown in Figure 3. A strong Al_2_O_3_ (0001) peak was observed at approximately 41.6° from the sapphire substrate. Typically, the 2θ peak of amorphous IZTO is broadly distributed between 20° and 35°, depending on its composition [20]. All samples exhibited a broad peak centered around 32° in the 2θ range of 20–60°, consistent with the amorphous nature of IZTO. Although the thermal annealing process is primarily intended to activate the channel layer, it is critical to confirm that the a-IZTO for TCEs, as well as for the channel, remains amorphous even after thermal annealing, particularly since the a-IZTO used as the bottom gate electrode undergoes the same thermal treatment as the TCE beneath the channel layer. The XRD results demonstrated no changes in the full width at half maximum (FWHM) and in the intensity of the observed peaks after annealing, indicating that no phase transition or crystallization occurred. This confirms that both the channel and TCE layers retained their amorphous states post-treatment.

**Figure 4:**
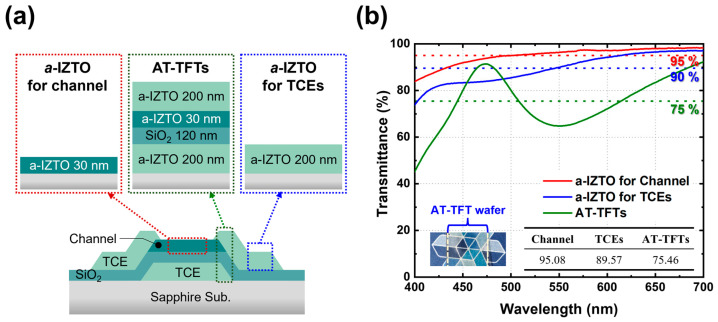
(**a**) Schematic image of the AT-TFT, showing the film thickness and layer structure when measuring the transmittance of a-IZTO for the channel, AT-TFT, and a-IZTO for TCEs. (**b**) Transmittance results for a-IZTO in the channel, AT-TFT, and a-IZTO for TCEs. The inset table shows the average transmittance in the 400–700 nm range.

To evaluate the optical transparency of the AT-TFT device, we measured the transmittance of each a-IZTO thin film used in the TCE and channel layers, as well as the transmittance of the entire multilayer structure constituting the device. Figure 4a illustrates the AT-TFT device structure and the specific configurations of each sample used for transmittance measurement. The a-IZTO film for the channel layer was fabricated as a 30 nm-thick single layer, while the TCE layer consisted of a 200 nm-thick a-IZTO film. The transmittance of the AT-TFT device was measured in regions where the largest number of thin films were deposited upon device completion. Specifically, the AT-TFT transmittance sample comprised a multilayer structure with 200 nm-thick a-IZTO, a 120 nm-thick SiO_2_ insulating layer, and an additional 230 nm-thick a-IZTO layer, as illustrated in Figure 4a.

The transmittance measurements for each configuration are shown in Figure 4b. The a-IZTO thin film for the channel layer demonstrated an average transmittance of 95.08%, and the a-IZTO film for the TCE exhibited an average transmittance of 89.57% in the visible light range (400–700 nm). These results confirm that the a-IZTO films used for both the channel and TCE layers achieved high transmittance, exceeding 90% in the visible spectrum. Meanwhile, the AT-TFT multilayer structure exhibited an average transmittance of 75.46% over the same wavelength range. The observed oscillations in the transmittance spectrum of the AT-TFT multilayer sample were attributed to interference effects caused by the multilayer structure. Additionally, the reduced average transmittance of the AT-TFT multilayer was primarily influenced by the thicker a-IZTO layers and the inclusion of the SiO_2_ insulating layer. However, as shown in the AT-TFT device structure in Figure 4a, the area featuring this multilayer configuration was limited in the actual device. The remaining regions, composed of thinner films, were expected to exhibit higher overall transmittance in practical applications. This analysis highlights the potential of a-IZTO films for high transparency, suitable for advanced transparent electronic devices.

**Figure 5:**
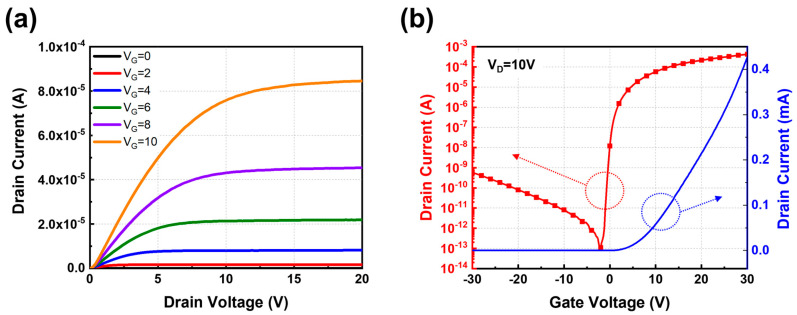
(**a**) The output and (**b**) transfer characteristics of AT-TFTs using all-amorphous oxide materials, including dual-functional a-IZTO films for channel and TCEs. The red line represents the drain current on a logarithmic scale, plotted on the left y-axis, while the blue line shows the linear measurement of the drain current, plotted on the right y-axis.

Figure 5a shows the output characteristics of AT-TFT devices, demonstrating that the drain current increases linearly with the drain voltage within a gate voltage range of 0–10 V. Furthermore, distinct pinch-off voltages and current saturation are observed at higher drain voltages. These results indicate excellent electrical ohmic contact between the source and drain, affirming that the thin-film transistor conforms to the expected characteristics of a field-effect transistor. Figure 5b shows the transfer characteristics of the AT-TFTs, and Table 1 provides a comprehensive summary of the TFT characteristics. The fabricated AT-TFTs demonstrate an impressive field-effect mobility of up to 30.12 cm^2^/V·s, along with an outstanding on/off current ratio exceeding 10^8^. This field-effect mobility is comparable to that of TFTs with an AOS channel layer, according to previous studies [21,22,23,24,25]. Additionally, to validate the photostability of the AT-TFT devices, transfer characteristics under positive bias illumination stress (PBIS) and negative bias illumination stress (NBIS) were measured under white light irradiation, as shown in Figure S2. Table S3 summarizes the threshold voltage values and their changes under different conditions. Under PBIS, the threshold voltage increased with the duration of the stress. In contrast, under NBIS, the threshold voltage shifted negatively with increasing bias stress duration. The generation of photo-induced carriers under white light irradiation led to a smaller positive shift in threshold voltage under PBIS compared to the negative shift observed under NBIS [26].

## 4. Conclusions

In this study, we fabricated AT-TFTs using only amorphous oxide materials to achieve high performance for flexible and transparent display applications. a-IZTO, which exhibits superior field-effect mobility compared to the widely studied a-IGZO, was employed as the channel layer. Additionally, to overcome the transparency degradation of conventional metal electrodes and the flexibility limitations of crystalline TCEs, a-IZTO was also applied to the source, drain, and gate electrodes. The dual-functional a-IZTO, serving as both the channel and TCE layers, was deposited via sputtering, and the roles of a-IZTO were controlled by composition variation. The a-IZTO for TCEs demonstrated excellent flexibility with a low sheet resistance of 18.13 Ω/sq. After 50,000 and 100,000 bending cycles, the sheet resistance changed by only 4.04% and 24.59%, respectively, confirming its mechanical robustness. To activate dopants in the channel layer, the fabricated AT-TFTs underwent post-annealing processes. XRD analysis confirmed that both the channel and TCE layers maintained their amorphous structure even after thermal annealing. Despite the incorporation of four multilayer films, the AT-TFT devices achieved an average transmittance of 75.46% in the visible light range. The completed AT-TFTs exhibited excellent field-effect transistor characteristics, including a high field-effect mobility of 30.12 cm^2^/V·s and an impressive on/off current ratio of 10^8^. Regarding photo-bias stability, the changes in threshold voltage induced by white light irradiation, particularly under NBIS conditions, were not negligible. Although this study primarily focused on TCEs, optimizing the channel materials and improving the device structure design present substantial potential for further enhancing the performance and stability of AT-TFT devices. These results demonstrate the potential applicability of the AT-TFTs to flexible and transparent display technologies.

## Figures and Tables

**Table 1:** Characteristics of a-IZTO AT-TFTs.

Field-Effect Mobility (cm^2^/V∙s)	Threshold Voltage (Vth)	On/Off Ratio	S.S (V/dec)	I_ds_ MAX (A)
30.12	0.87	~10^9^	0.36	4.28 × 10^−4^

## Data Availability

The original contributions presented in this study are included in the article/Supplementary Materials. Further inquiries can be directed to the corresponding author.

## Supplementary Materials

The following supporting information can be downloaded at: https://www.mdpi.com/article/10.3390/ma18020216/s1, Table S1: The metal composition of a-IZTO for the channel layer analyzed using SEM-EDS; Table S2: Sheet resistance and resistance changes of a-IZTO TCEs under bending tests; Figure S1: (a) The output and (b) transfer characteristics of AT-TFTs using all-amorphous oxide materials, including dual-functional a-IZTO films for channel and TCEs; Figure S2: Transfer characteristics of AT-TFTs under (a) positive bias illumination stress (PBIS) and (b) negative bias illumination stress (NBIS). The light exposure was conducted using white light irradiation; Table S3: The threshold voltages and the changes of threshold voltage for AT-TFTs under PBIS and NBIS.

## References

[1] Koo J.H., Kim D.C., Shim H.J., Kim T.H., Kim D.H. (2018). Flexible and stretchable smart display: Materials, fabrication, device design, and system integration. Adv. Funct. Mater..

[2] Shim G.W., Hong W., Cha J.H., Park J.H., Lee K.J., Choi S.Y. (2020). TFT channel materials for display applications: From amorphous silicon to transition metal dichalcogenides. Adv. Mater..

[3] Kamiya T., Hosono H. (2010). Material characteristics and applications of transparent amorphous oxide semiconductors. NPG Asia Mater..

[4] Zhu Y., He Y., Jiang S., Zhu L., Chen C., Wan Q. (2021). Indium–gallium–zinc–oxide thin-film transistors: Materials, devices, and applications. J. Semicond..

[5] Kamiya T., Nomura K., Hosono H. (2010). Present status of amorphous In–Ga–Zn–O thin-film transistors. Sci. Technol. Adv. Mater..

[6] Pan Z., Hu Y., Chen J., Wang F., Jeong Y., Pham D.P., Yi J. (2024). Approaches to Improve Mobility and Stability of IGZO TFTs: A Brief Review. Trans. Electr. Electron. Mater..

[7] Fuh C.-S., Liu P.-T., Huang W.-H., Sze S.M. (2014). Effect of annealing on defect elimination for high mobility amorphous indium-zinc-tin-oxide thin-film transistor. IEEE Electron Device Lett..

[8] Liu P.-T., Chang C.-H., Fuh C.-S. (2016). Enhancement of reliability and stability for transparent amorphous indium-zinc-tin-oxide thin film transistors. RSC Adv..

[9] Liu H., Avrutin V., Izyumskaya N., Özgür Ü., Morkoç H. (2010). Transparent conducting oxides for electrode applications in light emitting and absorbing devices. Superlattices Microstruct..

[10] Gwamuri J., Vora A., Khanal R.R., Phillips A.B., Heben M.J., Guney D.O., Bergstrom P., Kulkarni A., Pearce J.M. (2015). Limitations of ultra-thin transparent conducting oxides for integration into plasmonic-enhanced thin-film solar photovoltaic devices. Mater. Renew. Sustain. Energy.

[11] Axelevitch A., Gorenstein B., Golan G. (2012). Investigation of optical transmission in thin metal films. Phys. Procedia.

[12] Shi X.H., Xu K.J. (2017). Properties of fluorine-doped tin oxide films prepared by an improved sol-gel process. Mater. Sci. Semicond. Process..

[13] Song S., Yang T., Liu J., Xin Y., Li Y., Han S. (2011). Rapid thermal annealing of ITO films. Appl. Surf. Sci..

[14] Kerkache L., Layadi A., Dogheche E., Remiens D. (2005). Physical properties of RF sputtered ITO thin films and annealing effect. J. Phys. D Appl. Phys..

[15] Gonçalves G., Elangovan E., Barquinha P., Pereira L., Martins R., Fortunato E. (2007). Influence of post-annealing temperature on the properties exhibited by ITO, IZO and GZO thin films. Thin Solid Films.

[16] Medvedeva J.E., Buchholz D.B., Chang R.P.H. (2017). Recent advances in understanding the structure and properties of amorphous oxide semiconductors. Adv. Electron. Mater..

[17] Hosono H. (2006). Ionic amorphous oxide semiconductors: Material design, carrier transport, and device. Appl. J. Non-Cryst. Solids.

[18] Kim Y.S., Hwang W.J., Eun K.T., Choa S.-H. (2011). Mechanical reliability of transparent conducting IZTO film electrodes for flexible panel displays. Appl. Surf. Sci..

[19] Kwon J.H., Park J., Lee M.K., Park J.W., Jeon Y., Shin J.B., Nam M., Kim C.-K., Choi Y.-K., Choi K.C. (2018). Low-temperature fabrication of robust, transparent, and flexible thin-film transistors with a nanolaminated insulator. ACS Appl. Mater. Interfaces.

[20] Kim D.-H., Rim Y.-S., Kim K.-H., Son I.-H. (2009). Properties of IZTO Thin Films Prepared by Using a Hetero-Target Sputtering System. J. Korean Phys. Soc..

[21] Ji X., Yuan Y., Yin X., Yan S., Xin Q., Song A. (2022). High-Performance Thin-Film Transistors With Sputtered IGZO/Ga_2_O_3_ Heterojunction. IEEE Trans. Electron Devices.

[22] Su J., Yang H., Yang W., Zhang X. (2022). Electrical characteristics of tungsten-doped InZnSnO thin film transistors by RF magnetron sputtering. J. Vac. Sci. Technol. B.

[23] Yang H., Yang W., Su J., Zhang X. (2022). Enhancement-mode thin film transistor using amorphous phosphorus-doped Indium–Zinc–Tin-Oxide channel layer. Mater. Sci. Semicond. Process..

[24] Yan X., Li B., Song K., Zhang Y., Wang Y., Yang F., Wang C., Chi Y., Yang X. (2022). Fabrication and properties of InGaZnO thin-film transistors based on a sol–gel method with different electrode patterns. Micromachines.

[25] Park S.-J., Park S.-R., Na J.M., Jeon W.-S., Kang Y., Ham S., Kim Y.-H., Chung Y.-B., Ha T.-J. (2024). Charge transport properties of high-mobility indium-gallium-zinc oxide thin-film transistors fabricated through atomic-layer deposition. J. Mater. Chem. C.

[26] Jing B., Peng C., Xu M., Huang H., Li X., Zhang J. (2022). Investigation on Stability in Solution-Processed In-Zn-Sn-O TFT Array Under Various Intensity of Illumination. IEEE Trans. Electron Devices.

