# Supplementary material for: High-Mobility All-Transparent TFTs with Dual-Functional Amorphous IZTO for Channel and Transparent Conductive Electrodes
Source: Materials (Basel). 2025 Jan 7;18(2):216. doi: 10.3390/ma18020216 (PMC11766526; doi:10.3390/ma18020216)
Supplement: Supplementary file 1 [file materials-18-00216-s001.zip › materials-3380445-supplementary.pdf]

Supplementary Materials

High-Mobility All-Transparent TFTs with Dual-Functional Amorphous IZTO for Channel and Transparent Conductive Electrodes

Min-Woo Park [1,†], Sohyeon Kim [1,2,†], Su-Yeon Son [1], Si-Won Kim [1], Tae-Kyun Moon [1], Pei-Chen Su [3] and Kyoung-Kook Kim [1,2,*]

[1] Department of IT Semiconductor Convergence Engineering, Research Institute of Advanced Convergence Technology, Tech University of Korea, Siheung 15073, Republic of Korea

[2] Department of Nano & Semiconductor Engineering, Tech University of Korea, Siheung 15073, Republic of Korea

[3] School of Mechanical and Aerospace Engineering, Nanyang Technological University, 50 Nanyang Avenue, Singapore, 639798, Singapore

† These authors contributed equally to this work.

Table S1. The metal composition of a-IZTO for the channel layer analyzed using SEM-EDS.

Element	Zn	In	Sn	Totals
Atomic %	60.42	32.95	6.62	100.00

Table S2. Sheet resistance and resistance changes of a-IZTO TCEs under bending tests

	Bending cycle				
	0	10,000	30,000	50,000	100,000
Sheet resistance (ohm/sq)	18.13	18.24	18.26	18.86	22.65
Resistance change (%)	0	0.58	0.71	4.04	24.95

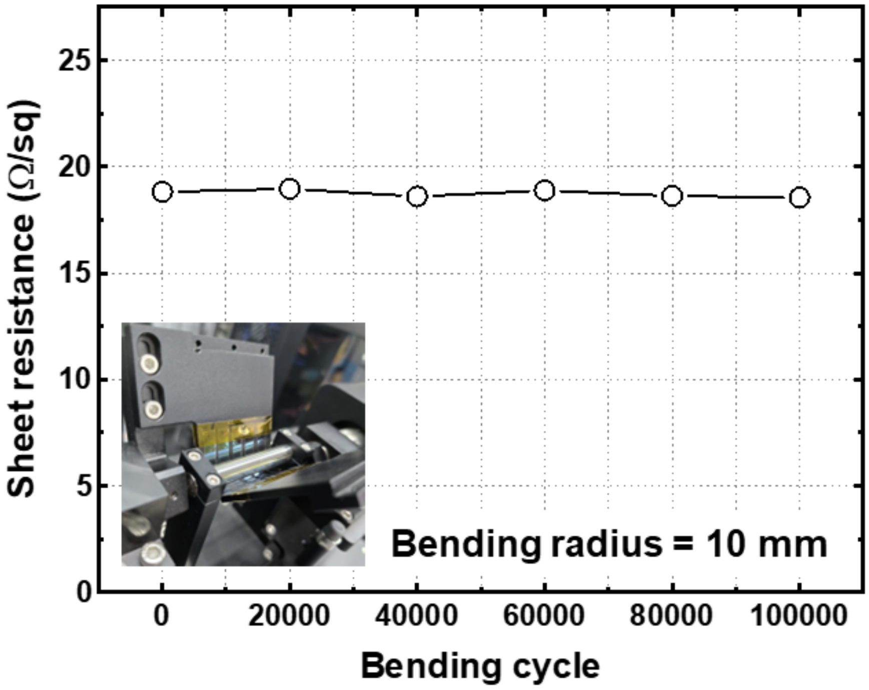

Figure S1. (a) The output and (b) transfer characteristics of AT-TFTs using all-amorphous oxide materials, including dual-functional a-IZTO films for channel and TCEs.

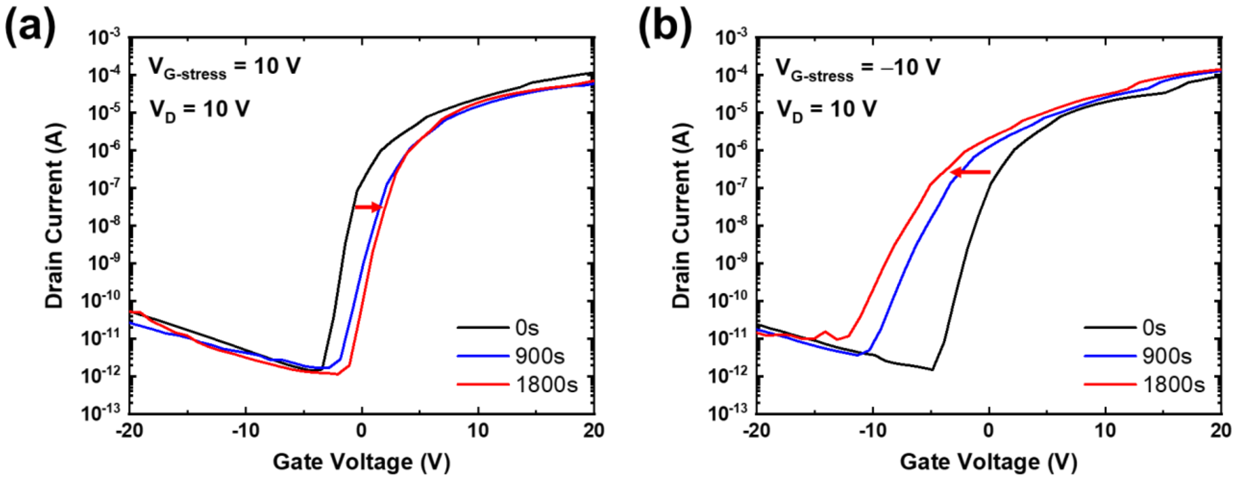

Figure S2. Transfer characteristics of AT-TFTs under (a) positive bias illumination stress (PBIS) and (b) negative bias illumination stress (NBIS). The light exposure was conducted using white light irradiation.

Table S3. The threshold voltages and the changes of threshold voltage for AT-TFTs under PBIS and NBIS.

	Stress duration	0s	900s	1800s
PBIS	V_{th}	0.82	0.91	1.15
	ΔV_{th}	-	0.09	0.33
NBIS	V_{th}	0.85	0.24	-1.24
	ΔV_{th}	-	-0.61	-2.09
